# Di-μ-iodido-bis­[(dimethyl 2,2′-biquinoline-4,4′-dicarboxyl­ate-κ^2^ *N*,*N*′)copper(I)]

**DOI:** 10.1107/S1600536812020843

**Published:** 2012-05-12

**Authors:** Radosław Starosta, Urszula K. Komarnicka, Justyna Nagaj, Kamila Stokowa-Sołtys, Aleksandra Bykowska

**Affiliations:** a Faculty of Chemistry, University of Wrocław, 14 Joliot-Curie St, 50-383 Wrocław, Poland

## Abstract

In the centrosymmetric dinuclear title complex, [Cu_2_I_2_(C_22_H_16_N_2_O_4_)_2_], the Cu^I^ atom is coordinated in a distorted tetra­hedral geometry by an *N*,*N*′-bidentate dimethyl 2,2′-biquinoline-4,4′-dicarboxyl­ate ligand and two symmetry-related I atoms, which act as bridges to a symmetry-related Cu^I^ atom. The distance between the Cu^I^ atoms within the dinuclear unit is 2.6723 (11) Å.

## Related literature

Copper(I) complexes are a subject of high inter­est and have been extensively studied during the past two decades because of their diversified photo-physical properties (Lavie-Cambot *et al.*, 2008 ▶; Vorontsov *et al.*, 2009 ▶; Hashimoto *et al.*, 2011 ▶). The title complex is similar to other copper(I) complexes with halides and aromatic diimines: [Cu_2_I_2_(1,10-phenanthroline)_2_] and Cu_2_ *X*_2_(2,9-dimethyl-1,10-phenanthroline)_2_], where *X* = I, Br, Cl (Healy *et al.*, 1985 ▶); [Cu_2_ *X*_2_(1,10-phenanthroline)_2_], where *X* = Cl and I (Yu *et al.*, 2004 ▶); [Cu_2_ *X*_2_(NN)_2_], where *X* = Br, I and NN = bidentate imino nitroxides (Oshio *et al.*, 1996 ▶); [Cu_2_Cl_2_(dihexsyl-2,2′-biquinoline-4,4′-dicarboxyl­ate)_2_] [Cu_2_Cl_2_(2,2′-biquinoline-4,4′-dicarb­oxy­lic acid)_2_] (Vatsadze *et al.*, 2010 ▶). For the preparation of the dimethyl-2,2′-biquinoline-4,4′-dicarboxyl­ate ligand, see: Pucci *et al.* (2011 ▶) and of the P(CH_2_N(CH_2_CH_2_)_2_O)_3_ phosphane ligand, see: Starosta *et al.* (2010 ▶).
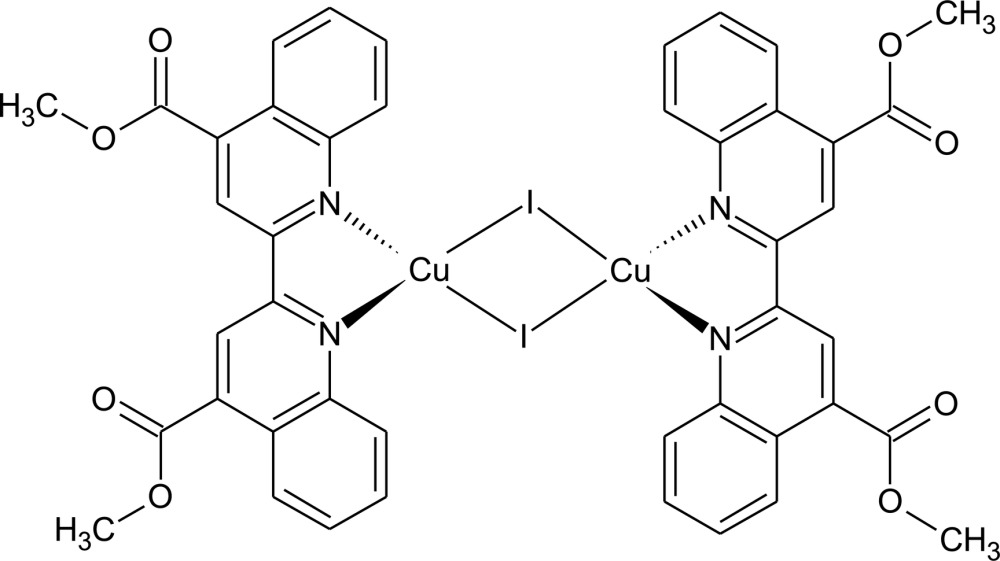


## Experimental

### 

#### Crystal data

[Cu_2_I_2_(C_22_H_16_N_2_O_4_)_2_]
*M* *_r_* = 1125.62
Triclinic, 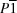

*a* = 8.792 (3) Å
*b* = 9.157 (3) Å
*c* = 12.865 (4) Å
α = 96.59 (3)°
β = 102.49 (3)°
γ = 103.51 (3)°
*V* = 968.2 (5) Å^3^
*Z* = 1
Mo *K*α radiation
μ = 2.76 mm^−1^
*T* = 100 K
0.15 × 0.10 × 0.10 mm

#### Data collection

Kuma KM-4-CCD κ-geometry diffractometer
Absorption correction: analytical [*CrysAlis RED* (Oxford Diffraction, 2006 ▶), based on expressions derived by Clark & Reid (1995 ▶)] *T*_min_ = 0.466, *T*_max_ = 0.912
15308 measured reflections
5471 independent reflections
4606 reflections with *I* > 2σ(*I*)
*R*_int_ = 0.028

#### Refinement

*R*[*F* ^2^ > 2σ(*F* ^2^)] = 0.027
*wR*(*F* ^2^) = 0.065
*S* = 1.02
5471 reflections
273 parameters
H-atom parameters constrained
Δρ_max_ = 0.89 e Å^−3^
Δρ_min_ = −1.16 e Å^−3^

### 

Data collection: *CrysAlis CCD* (Oxford Diffraction, 2006 ▶); cell refinement: *CrysAlis RED* (Oxford Diffraction, 2006 ▶); data reduction: *CrysAlis RED*; program(s) used to solve structure: *SHELXS97* (Sheldrick, 2008 ▶); program(s) used to refine structure: *SHELXL97* (Sheldrick, 2008 ▶); molecular graphics: *Mercury* (Macrae *et al.*, 2006 ▶); software used to prepare material for publication: *publCIF* (Westrip, 2010 ▶).

## Supplementary Material

Crystal structure: contains datablock(s) I, global. DOI: 10.1107/S1600536812020843/kp2406sup1.cif

Structure factors: contains datablock(s) I. DOI: 10.1107/S1600536812020843/kp2406Isup2.hkl

Additional supplementary materials:  crystallographic information; 3D view; checkCIF report

## Figures and Tables

**Table 1:** Selected bond lengths (Å)

Cu1—N1*A*	2.088 (2)
Cu1—N1*B*	2.092 (2)
Cu1—I1	2.5473 (10)
Cu1—I1^i^	2.6996 (9)

Symmetry code: (i) 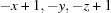.

**Fig. 1.**
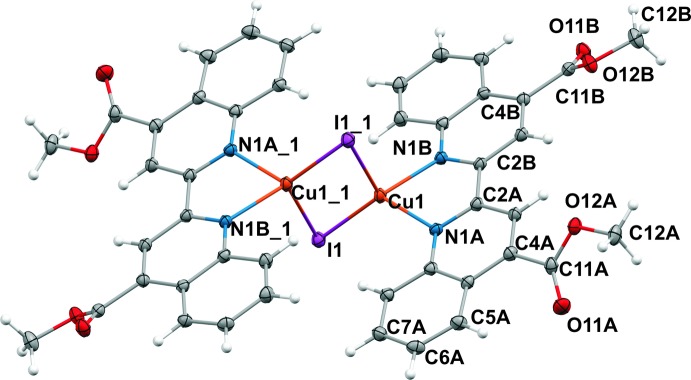
The molecular structure of the complex showing the atom-labelling scheme and displacement ellipsoids at the 50% probability (symmmetry code used: -x + 1, -y, -z + 1).

## supplementary crystallographic information

### Comment

The asymmetric unit of the studied bis((µ-iodo)-(dimethyl-2,2'-biquinoline-4,4'-dicarboxylate))-di-copper(I) complex consist of the [(dimethyl-2,2'-biquinoline-4,4'-dicarboxylate)Cu(I)] moiety (Fig. 1, Table 1). Cu^I^ atoms are bridged by two iodide ions forming the planar rhombic Cu_2_(µ-I)_2_ core. Additionally coordinated by the imine nitrogen atoms of the dimethyl-2,2'-biquinoline-4,4'-dicarboxylate ligand, each Cu^I^ atom reveals a distorted tetrahedral geometry. Connected quinoline rings of the coordinated molecule of dimethyl-2,2'-biquinoline-4,4'-dicarboxylate are not coplanar, the angle between their planes is 5.40 (7)°.

### Experimental

Crystals of the title complex were grown in the mixture of dichloromethane and acetone in an attempt to obtain crystals of [Cu(I)(dimethyl-2,2'-biquinoline-4,4'-dicarboxylate) P(CH_2_N(CH_2_CH_2_)_2_O)_3_] complex. Cu^I^ was purchased from Aldrich. Dimethyl-2,2'-biquinoline-4,4'- dicarboxylate ligand was prepared from 2,2'-biquinoline-4,4'-dicarboxylic acid (Aldrich) according to the literature method (Pucci *et al.*, 2011). P(CH_2_N(CH_2_CH_2_)_2_O)_3_ phosphane ligand was synthesized as described previously (Starosta *et al.*, 2010).

### Refinement

All hydrogen atoms were placed in geometrically idealized positions and constrained to ride on their parent atoms.

### Crystal data

**Table:**

[Cu_2_I_2_(C_22_H_16_N_2_O_4_)_2_]	*Z* = 1
*M**_r_* = 1125.62	*F*(000) = 552
Triclinic, *P*1	*D*_x_ = 1.930 Mg m^−^^3^
Hall symbol: -P 1	Mo *K*α radiation, λ = 0.71073 Å
*a* = 8.792 (3) Å	Cell parameters from 11359 reflections
*b* = 9.157 (3) Å	θ = 2.9–36.8°
*c* = 12.865 (4) Å	µ = 2.76 mm^−^^1^
α = 96.59 (3)°	*T* = 100 K
β = 102.49 (3)°	Plate, orange
γ = 103.51 (3)°	0.15 × 0.10 × 0.10 mm
*V* = 968.2 (5) Å^3^	

### Data collection

**Table:**

Kuma KM-4-CCD κ-geometry diffractometer	5471 independent reflections
Radiation source: fine-focus sealed tube	4606 reflections with *I* > 2σ(*I*)
Graphite monochromator	*R*_int_ = 0.028
ω scans	θ_max_ = 30.0°, θ_min_ = 2.9°
Absorption correction: analytical [*CrysAlis RED* (Oxford Diffraction, 2006), based on expressions derived by Clark & Reid (1995)]	*h* = −10→12
*T*_min_ = 0.466, *T*_max_ = 0.912	*k* = −12→11
15308 measured reflections	*l* = −17→17

### Refinement

**Table:**

Refinement on *F*^2^	Primary atom site location: structure-invariant direct methods
Least-squares matrix: full	Secondary atom site location: difference Fourier map
*R*[*F*^2^ > 2σ(*F*^2^)] = 0.027	Hydrogen site location: inferred from neighbouring sites
*wR*(*F*^2^) = 0.065	H-atom parameters constrained
*S* = 1.02	*w* = 1/[σ^2^(*F*_o_^2^) + (0.040*P*)^2^] where *P* = (*F*_o_^2^ + 2*F*_c_^2^)/3
5471 reflections	(Δ/σ)_max_ = 0.001
273 parameters	Δρ_max_ = 0.89 e Å^−^^3^
0 restraints	Δρ_min_ = −1.16 e Å^−^^3^

### Special details

**Table:**

Experimental. Absorption correction: CrysAlis RED, (Oxford Diffraction, 2006). Analytical numeric absorption correction using a multifaceted crystal model based on expressions derived by R.C. Clark & J.S. Reid. (Clark, R. C. & Reid, J. S., 1995)
Geometry. All e.s.d.'s (except the e.s.d. in the dihedral angle between two l.s. planes) are estimated using the full covariance matrix. The cell e.s.d.'s are taken into account individually in the estimation of e.s.d.'s in distances, angles and torsion angles; correlations between e.s.d.'s in cell parameters are only used when they are defined by crystal symmetry. An approximate (isotropic) treatment of cell e.s.d.'s is used for estimating e.s.d.'s involving l.s. planes.
Refinement. Refinement of *F*^2^ against ALL reflections. The weighted *R*-factor *wR* and goodness of fit *S* are based on *F*^2^, conventional *R*-factors *R* are based on *F*, with *F* set to zero for negative *F*^2^. The threshold expression of *F*^2^ > σ(*F*^2^) is used only for calculating *R*-factors(gt) *etc*. and is not relevant to the choice of reflections for refinement. *R*-factors based on *F*^2^ are statistically about twice as large as those based on *F*, and *R*- factors based on ALL data will be even larger.

### Fractional atomic coordinates and isotropic or equivalent isotropic displacement parameters (Å^2^)

**Table:**

	*x*	*y*	*z*	*U*_iso_*/*U*_eq_	
Cu1	0.34262 (3)	−0.02511 (3)	0.49564 (2)	0.01649 (7)	
I1	0.495174 (17)	0.240323 (17)	0.478225 (12)	0.01826 (5)	
N1A	0.1380 (2)	−0.1629 (2)	0.38141 (15)	0.0142 (3)	
C2A	0.0293 (3)	−0.2436 (3)	0.42433 (18)	0.0153 (4)	
C3A	−0.0976 (3)	−0.3688 (3)	0.36264 (18)	0.0172 (4)	
H3A	−0.1715	−0.4265	0.3963	0.021*	
C4A	−0.1132 (3)	−0.4064 (3)	0.25341 (18)	0.0165 (4)	
C5A	−0.0028 (3)	−0.3491 (3)	0.09277 (18)	0.0191 (4)	
H5A	−0.0859	−0.4295	0.0451	0.023*	
C6A	0.1133 (3)	−0.2621 (3)	0.05366 (19)	0.0204 (5)	
H6A	0.1111	−0.2840	−0.0207	0.024*	
C7A	0.2367 (3)	−0.1399 (3)	0.12198 (19)	0.0200 (5)	
H7A	0.3155	−0.0795	0.0932	0.024*	
C8A	0.2426 (3)	−0.1086 (3)	0.22991 (19)	0.0178 (4)	
H8A	0.3256	−0.0265	0.2759	0.021*	
C9A	0.1252 (3)	−0.1986 (2)	0.27237 (18)	0.0150 (4)	
C10A	−0.0005 (3)	−0.3205 (3)	0.20414 (18)	0.0158 (4)	
C11A	−0.2508 (3)	−0.5363 (3)	0.18690 (19)	0.0190 (4)	
O11A	−0.3182 (2)	−0.5417 (2)	0.09388 (14)	0.0281 (4)	
O12A	−0.28999 (19)	−0.64555 (19)	0.24393 (14)	0.0212 (3)	
C12A	−0.4228 (3)	−0.7757 (3)	0.1849 (2)	0.0245 (5)	
H12D	−0.5155	−0.7399	0.1519	0.037*	
H12E	−0.4536	−0.8435	0.2349	0.037*	
H12F	−0.3887	−0.8313	0.1283	0.037*	
N1B	0.1763 (2)	−0.0732 (2)	0.58953 (15)	0.0144 (3)	
C2B	0.0491 (2)	−0.1915 (3)	0.54192 (17)	0.0140 (4)	
C3B	−0.0588 (3)	−0.2629 (3)	0.59879 (18)	0.0154 (4)	
H3B	−0.1447	−0.3501	0.5632	0.019*	
C4B	−0.0395 (3)	−0.2061 (3)	0.70607 (18)	0.0155 (4)	
C5B	0.1212 (3)	−0.0045 (3)	0.86815 (18)	0.0169 (4)	
H5B	0.0512	−0.0429	0.9113	0.020*	
C6B	0.2517 (3)	0.1184 (3)	0.91204 (18)	0.0193 (4)	
H6B	0.2709	0.1642	0.9854	0.023*	
C7B	0.3583 (3)	0.1782 (3)	0.85011 (19)	0.0188 (4)	
H7B	0.4489	0.2630	0.8821	0.023*	
C8B	0.3309 (3)	0.1139 (3)	0.74412 (18)	0.0176 (4)	
H8B	0.4023	0.1548	0.7025	0.021*	
C9B	0.1967 (3)	−0.0135 (3)	0.69604 (18)	0.0153 (4)	
C10B	0.0894 (3)	−0.0752 (2)	0.75828 (17)	0.0145 (4)	
C11B	−0.1583 (3)	−0.2831 (3)	0.76375 (18)	0.0163 (4)	
O11B	−0.1970 (2)	−0.2197 (2)	0.83756 (14)	0.0220 (4)	
O12B	−0.2166 (2)	−0.43175 (19)	0.72286 (14)	0.0210 (3)	
C12B	−0.3430 (3)	−0.5141 (3)	0.7667 (2)	0.0241 (5)	
H12A	−0.3126	−0.4851	0.8456	0.036*	
H12B	−0.3575	−0.6240	0.7470	0.036*	
H12C	−0.4443	−0.4890	0.7371	0.036*	

### Atomic displacement parameters (Å^2^)

**Table:**

	*U*^11^	*U*^22^	*U*^33^	*U*^12^	*U*^13^	*U*^23^
Cu1	0.01350 (13)	0.01802 (14)	0.01445 (13)	−0.00091 (10)	0.00308 (10)	0.00031 (10)
I1	0.01682 (7)	0.01604 (7)	0.01965 (8)	0.00169 (5)	0.00320 (5)	0.00222 (5)
N1A	0.0140 (8)	0.0151 (9)	0.0116 (8)	0.0030 (7)	0.0011 (7)	0.0007 (7)
C2A	0.0139 (9)	0.0167 (10)	0.0136 (10)	0.0032 (8)	0.0028 (8)	−0.0001 (8)
C3A	0.0139 (10)	0.0188 (11)	0.0161 (10)	0.0003 (8)	0.0029 (8)	0.0020 (8)
C4A	0.0170 (10)	0.0145 (10)	0.0150 (10)	0.0019 (8)	0.0020 (8)	−0.0011 (8)
C5A	0.0216 (11)	0.0190 (11)	0.0140 (10)	0.0038 (9)	0.0028 (8)	−0.0011 (8)
C6A	0.0274 (12)	0.0200 (11)	0.0122 (10)	0.0059 (9)	0.0039 (9)	−0.0006 (8)
C7A	0.0227 (11)	0.0205 (11)	0.0170 (10)	0.0040 (9)	0.0063 (9)	0.0051 (9)
C8A	0.0173 (10)	0.0180 (11)	0.0160 (10)	0.0026 (8)	0.0023 (8)	0.0027 (8)
C9A	0.0145 (9)	0.0146 (10)	0.0142 (10)	0.0028 (8)	0.0018 (8)	0.0016 (8)
C10A	0.0150 (10)	0.0160 (10)	0.0140 (10)	0.0031 (8)	0.0009 (8)	−0.0001 (8)
C11A	0.0157 (10)	0.0198 (11)	0.0186 (11)	0.0011 (8)	0.0053 (8)	−0.0024 (9)
O11A	0.0260 (9)	0.0315 (10)	0.0161 (8)	−0.0037 (8)	−0.0016 (7)	−0.0013 (7)
O12A	0.0164 (8)	0.0178 (8)	0.0224 (8)	−0.0030 (6)	0.0000 (6)	0.0000 (7)
C12A	0.0162 (11)	0.0184 (11)	0.0312 (13)	−0.0030 (9)	0.0012 (10)	−0.0017 (10)
N1B	0.0129 (8)	0.0160 (9)	0.0123 (8)	0.0013 (7)	0.0024 (7)	0.0008 (7)
C2B	0.0121 (9)	0.0148 (10)	0.0124 (9)	0.0012 (7)	0.0013 (7)	0.0002 (8)
C3B	0.0118 (9)	0.0170 (10)	0.0151 (10)	0.0016 (8)	0.0012 (8)	0.0016 (8)
C4B	0.0129 (9)	0.0173 (10)	0.0158 (10)	0.0034 (8)	0.0039 (8)	0.0021 (8)
C5B	0.0176 (10)	0.0192 (11)	0.0138 (10)	0.0056 (8)	0.0036 (8)	0.0022 (8)
C6B	0.0220 (11)	0.0211 (11)	0.0124 (10)	0.0053 (9)	0.0024 (8)	−0.0020 (8)
C7B	0.0175 (10)	0.0162 (10)	0.0180 (10)	0.0004 (8)	0.0018 (8)	−0.0021 (8)
C8B	0.0167 (10)	0.0181 (11)	0.0157 (10)	0.0007 (8)	0.0047 (8)	0.0005 (8)
C9B	0.0141 (9)	0.0168 (10)	0.0142 (10)	0.0042 (8)	0.0023 (8)	0.0011 (8)
C10B	0.0142 (9)	0.0157 (10)	0.0135 (10)	0.0042 (8)	0.0030 (8)	0.0021 (8)
C11B	0.0145 (9)	0.0182 (10)	0.0149 (10)	0.0035 (8)	0.0017 (8)	0.0036 (8)
O11B	0.0212 (8)	0.0233 (9)	0.0191 (8)	0.0007 (7)	0.0085 (7)	−0.0016 (7)
O12B	0.0214 (8)	0.0167 (8)	0.0250 (9)	0.0013 (6)	0.0110 (7)	0.0025 (7)
C12B	0.0244 (12)	0.0179 (11)	0.0299 (13)	0.0001 (9)	0.0132 (10)	0.0042 (10)

### Geometric parameters (Å, º)

**Table:**

Cu1—N1A	2.088 (2)	C12A—H12D	0.9800
Cu1—N1B	2.092 (2)	C12A—H12E	0.9800
Cu1—I1	2.5473 (10)	C12A—H12F	0.9800
Cu1—I1^i^	2.6996 (9)	N1B—C2B	1.336 (3)
Cu1—Cu1^i^	2.6723 (11)	N1B—C9B	1.373 (3)
I1—Cu1^i^	2.6997 (9)	C2B—C3B	1.408 (3)
N1A—C2A	1.325 (3)	C3B—C4B	1.377 (3)
N1A—C9A	1.377 (3)	C3B—H3B	0.9500
C2A—C3A	1.414 (3)	C4B—C10B	1.422 (3)
C2A—C2B	1.493 (3)	C4B—C11B	1.500 (3)
C3A—C4A	1.377 (3)	C5B—C6B	1.368 (3)
C3A—H3A	0.9500	C5B—C10B	1.425 (3)
C4A—C10A	1.423 (3)	C5B—H5B	0.9500
C4A—C11A	1.502 (3)	C6B—C7B	1.413 (3)
C5A—C6A	1.366 (3)	C6B—H6B	0.9500
C5A—C10A	1.421 (3)	C7B—C8B	1.368 (3)
C5A—H5A	0.9500	C7B—H7B	0.9500
C6A—C7A	1.412 (3)	C8B—C9B	1.419 (3)
C6A—H6A	0.9500	C8B—H8B	0.9500
C7A—C8A	1.372 (3)	C9B—C10B	1.426 (3)
C7A—H7A	0.9500	C11B—O11B	1.208 (3)
C8A—C9A	1.412 (3)	C11B—O12B	1.336 (3)
C8A—H8A	0.9500	O12B—C12B	1.448 (3)
C9A—C10A	1.420 (3)	C12B—H12A	0.9800
C11A—O11A	1.205 (3)	C12B—H12B	0.9800
C11A—O12A	1.332 (3)	C12B—H12C	0.9800
O12A—C12A	1.455 (3)		
			
N1A—Cu1—N1B	78.10 (8)	O12A—C12A—H12F	109.5
N1A—Cu1—I1	124.55 (6)	H12D—C12A—H12F	109.5
N1B—Cu1—I1	125.61 (6)	H12E—C12A—H12F	109.5
N1A—Cu1—I1^i^	96.95 (6)	C2B—N1B—C9B	118.65 (19)
N1B—Cu1—I1^i^	103.46 (6)	C2B—N1B—Cu1	113.28 (14)
I1—Cu1—I1^i^	118.85 (3)	C9B—N1B—Cu1	127.25 (15)
Cu1—I1—Cu1^i^	61.15 (3)	N1B—C2B—C3B	122.3 (2)
C2A—N1A—C9A	119.28 (19)	N1B—C2B—C2A	115.52 (19)
C2A—N1A—Cu1	113.75 (15)	C3B—C2B—C2A	122.14 (19)
C9A—N1A—Cu1	125.41 (15)	C4B—C3B—C2B	119.9 (2)
N1A—C2A—C3A	122.3 (2)	C4B—C3B—H3B	120.1
N1A—C2A—C2B	115.21 (19)	C2B—C3B—H3B	120.1
C3A—C2A—C2B	122.5 (2)	C3B—C4B—C10B	119.5 (2)
C4A—C3A—C2A	119.4 (2)	C3B—C4B—C11B	118.5 (2)
C4A—C3A—H3A	120.3	C10B—C4B—C11B	121.9 (2)
C2A—C3A—H3A	120.3	C6B—C5B—C10B	120.6 (2)
C3A—C4A—C10A	119.8 (2)	C6B—C5B—H5B	119.7
C3A—C4A—C11A	119.7 (2)	C10B—C5B—H5B	119.7
C10A—C4A—C11A	120.5 (2)	C5B—C6B—C7B	121.1 (2)
C6A—C5A—C10A	120.6 (2)	C5B—C6B—H6B	119.4
C6A—C5A—H5A	119.7	C7B—C6B—H6B	119.4
C10A—C5A—H5A	119.7	C8B—C7B—C6B	119.9 (2)
C5A—C6A—C7A	121.1 (2)	C8B—C7B—H7B	120.0
C5A—C6A—H6A	119.5	C6B—C7B—H7B	120.0
C7A—C6A—H6A	119.5	C7B—C8B—C9B	120.4 (2)
C8A—C7A—C6A	120.0 (2)	C7B—C8B—H8B	119.8
C8A—C7A—H7A	120.0	C9B—C8B—H8B	119.8
C6A—C7A—H7A	120.0	N1B—C9B—C8B	117.5 (2)
C7A—C8A—C9A	119.8 (2)	N1B—C9B—C10B	122.5 (2)
C7A—C8A—H8A	120.1	C8B—C9B—C10B	120.0 (2)
C9A—C8A—H8A	120.1	C4B—C10B—C5B	125.0 (2)
N1A—C9A—C8A	117.3 (2)	C4B—C10B—C9B	117.0 (2)
N1A—C9A—C10A	122.1 (2)	C5B—C10B—C9B	118.0 (2)
C8A—C9A—C10A	120.6 (2)	O11B—C11B—O12B	124.0 (2)
C9A—C10A—C5A	117.9 (2)	O11B—C11B—C4B	124.8 (2)
C9A—C10A—C4A	117.1 (2)	O12B—C11B—C4B	111.19 (19)
C5A—C10A—C4A	125.0 (2)	C11B—O12B—C12B	115.55 (18)
O11A—C11A—O12A	123.9 (2)	O12B—C12B—H12A	109.5
O11A—C11A—C4A	124.7 (2)	O12B—C12B—H12B	109.5
O12A—C11A—C4A	111.4 (2)	H12A—C12B—H12B	109.5
C11A—O12A—C12A	114.73 (19)	O12B—C12B—H12C	109.5
O12A—C12A—H12D	109.5	H12A—C12B—H12C	109.5
O12A—C12A—H12E	109.5	H12B—C12B—H12C	109.5
H12D—C12A—H12E	109.5		
			
N1A—Cu1—I1—Cu1^i^	−123.16 (7)	I1—Cu1—N1B—C2B	140.88 (14)
N1B—Cu1—I1—Cu1^i^	136.17 (7)	I1^i^—Cu1—N1B—C2B	−77.71 (15)
I1^i^—Cu1—I1—Cu1^i^	0.0	N1A—Cu1—N1B—C9B	−173.9 (2)
N1B—Cu1—N1A—C2A	−17.89 (15)	I1—Cu1—N1B—C9B	−49.7 (2)
I1—Cu1—N1A—C2A	−143.16 (14)	I1^i^—Cu1—N1B—C9B	91.69 (18)
I1^i^—Cu1—N1A—C2A	84.46 (15)	C9B—N1B—C2B—C3B	−4.1 (3)
N1B—Cu1—N1A—C9A	176.59 (19)	Cu1—N1B—C2B—C3B	166.34 (17)
I1—Cu1—N1A—C9A	51.33 (19)	C9B—N1B—C2B—C2A	176.24 (19)
I1^i^—Cu1—N1A—C9A	−81.05 (18)	Cu1—N1B—C2B—C2A	−13.4 (2)
C9A—N1A—C2A—C3A	1.9 (3)	N1A—C2A—C2B—N1B	−1.9 (3)
Cu1—N1A—C2A—C3A	−164.62 (17)	C3A—C2A—C2B—N1B	179.0 (2)
C9A—N1A—C2A—C2B	−177.27 (19)	N1A—C2A—C2B—C3B	178.4 (2)
Cu1—N1A—C2A—C2B	16.2 (2)	C3A—C2A—C2B—C3B	−0.7 (3)
N1A—C2A—C3A—C4A	−2.1 (3)	N1B—C2B—C3B—C4B	3.3 (3)
C2B—C2A—C3A—C4A	177.0 (2)	C2A—C2B—C3B—C4B	−177.0 (2)
C2A—C3A—C4A—C10A	0.9 (3)	C2B—C3B—C4B—C10B	0.2 (3)
C2A—C3A—C4A—C11A	−178.1 (2)	C2B—C3B—C4B—C11B	178.9 (2)
C10A—C5A—C6A—C7A	−1.2 (4)	C10B—C5B—C6B—C7B	−0.1 (4)
C5A—C6A—C7A—C8A	1.0 (4)	C5B—C6B—C7B—C8B	0.6 (4)
C6A—C7A—C8A—C9A	0.0 (3)	C6B—C7B—C8B—C9B	−0.5 (4)
C2A—N1A—C9A—C8A	179.3 (2)	C2B—N1B—C9B—C8B	−178.8 (2)
Cu1—N1A—C9A—C8A	−15.9 (3)	Cu1—N1B—C9B—C8B	12.3 (3)
C2A—N1A—C9A—C10A	−0.5 (3)	C2B—N1B—C9B—C10B	1.5 (3)
Cu1—N1A—C9A—C10A	164.31 (16)	Cu1—N1B—C9B—C10B	−167.44 (16)
C7A—C8A—C9A—N1A	179.4 (2)	C7B—C8B—C9B—N1B	−179.7 (2)
C7A—C8A—C9A—C10A	−0.8 (3)	C7B—C8B—C9B—C10B	0.0 (3)
N1A—C9A—C10A—C5A	−179.6 (2)	C3B—C4B—C10B—C5B	179.2 (2)
C8A—C9A—C10A—C5A	0.6 (3)	C11B—C4B—C10B—C5B	0.6 (3)
N1A—C9A—C10A—C4A	−0.6 (3)	C3B—C4B—C10B—C9B	−2.6 (3)
C8A—C9A—C10A—C4A	179.5 (2)	C11B—C4B—C10B—C9B	178.8 (2)
C6A—C5A—C10A—C9A	0.4 (3)	C6B—C5B—C10B—C4B	177.8 (2)
C6A—C5A—C10A—C4A	−178.5 (2)	C6B—C5B—C10B—C9B	−0.4 (3)
C3A—C4A—C10A—C9A	0.4 (3)	N1B—C9B—C10B—C4B	1.8 (3)
C11A—C4A—C10A—C9A	179.4 (2)	C8B—C9B—C10B—C4B	−177.9 (2)
C3A—C4A—C10A—C5A	179.3 (2)	N1B—C9B—C10B—C5B	−179.8 (2)
C11A—C4A—C10A—C5A	−1.7 (3)	C8B—C9B—C10B—C5B	0.4 (3)
C3A—C4A—C11A—O11A	146.9 (3)	C3B—C4B—C11B—O11B	−149.8 (2)
C10A—C4A—C11A—O11A	−32.1 (4)	C10B—C4B—C11B—O11B	28.8 (3)
C3A—C4A—C11A—O12A	−32.9 (3)	C3B—C4B—C11B—O12B	30.2 (3)
C10A—C4A—C11A—O12A	148.1 (2)	C10B—C4B—C11B—O12B	−151.2 (2)
O11A—C11A—O12A—C12A	0.2 (3)	O11B—C11B—O12B—C12B	5.1 (3)
C4A—C11A—O12A—C12A	179.96 (18)	C4B—C11B—O12B—C12B	−174.87 (19)
N1A—Cu1—N1B—C2B	16.69 (15)		

Symmetry code: (i) −*x*+1, −*y*, −*z*+1.
